# Relationships between Hematopoiesis and Hepatogenesis in the Midtrimester Fetal Liver Characterized by Dynamic Transcriptomic and Proteomic Profiles

**DOI:** 10.1371/journal.pone.0007641

**Published:** 2009-10-28

**Authors:** Yuanbiao Guo, Xuequn Zhang, Jian Huang, Yan Zeng, Wei Liu, Chao Geng, Ka Wan Li, Dong Yang, Songfeng Wu, Handong Wei, Zeguang Han, Xiaohong Qian, Ying Jiang, Fuchu He

**Affiliations:** 1 State Key Laboratory of Proteomics, Beijing Proteome Research Center, Beijing Institute of Radiation Medicine, Beijing, China; 2 Chinese National Human Genome Center at Shanghai, Shanghai, China; 3 Department of Gastroenterology, the First Affiliated Hospital, College of Medicine, Zhejiang University, Hangzhou, China; 4 Institutes of Biomedical Sciences, Fudan University, Shanghai, China; 5 Department of Molecular and Cellular Neurobiology, Center for Neurogenomics and Cognitive Research, Faculty of Earth and Life Sciences, Vrije Universiteit, De Boelelaan, Amsterdam, The Netherlands; 6 Medical Sciences Research Center, the Third People's Hospital of Chengdu, Chengdu, Sichuan, China; Beijing Institute of Infectious Diseases, China

## Abstract

In fetal hematopoietic organs, the switch from hematopoiesis is hypothesized to be a critical time point for organogenesis, but it is not yet evidenced. The transient coexistence of hematopoiesis will be useful to understand the development of fetal liver (FL) around this time and its relationship to hematopoiesis. Here, the temporal and the comparative transcriptomic and proteomic profiles were observed during the critical time points corresponding to the initiation (E11.5), peak (E14.5), recession (E15.5), and disappearance (3 ddp) of mouse FL hematopoiesis. We found that E11.5-E14.5 corresponds to a FL hematopoietic expansion phase with distinct molecular features, including the expression of new transcription factors, many of which are novel KRAB (Kruppel-associated box)-containing zinc finger proteins. This time period is also characterized by extensive depression of some liver functions, especially catabolism/utilization, immune and defense, classical complement cascades, and intrinsic blood coagulation. Instead, the other liver functions increased, such as xenobiotic and sterol metabolism, synthesis of carbohydrate and glycan, the alternate and lectin complement cascades and extrinsic blood coagulation, and etc. Strikingly, all of the liver functions were significantly increased at E14.5-E15.5 and thereafter, and the depression of the key pathways attributes to build the hematopoietic microenvironment. These findings signal hematopoiesis emigration is the key to open the door of liver maturation.

## Introduction

During the embryonic development of many species, hematopoiesis shifts among multiple anatomical sites, including the aorta-gonadmesonephros region (AGM), yolk sac (YS), placenta, fetal liver (FL), and bone marrow (BM). The shift in hematopoiesis from one location to another is attributable to changes in the local hematopoietic microenvironment that are initiated by embryonic organogenesis[1]. The switch in hematopoietic organs is thought to be a critical time point in embryogenesis and organogenesis, however, there are little evidences. FL is generally used as a model for developmental hematopoiesis[2]. The transient coexistence of hematopoiesis is useful to study the liver development. At such a hematopoiesis-prevailing stage and thereafter, the characteristics of liver development, particularly, a global view at the relationship between hematopoiesis and liver development, is little known.

Liver development in mice occurs in two main stages, with each having distinct biological properties and being separated by the time at which hematopoietic stem cells (HSCs) from the YS or AGM settle into the liver bud at around embryonic day 11 (E11)[3]. The first stage starts at around E8–9. During this time, the hepatic lineage arises from foregut endoderm in response to cardiac signals, and the liver bud is formed. The second stage probably begins at around E11. During this stage, the number of fetal liver HSCs (FL-HSCs) dramatically increases from 3 to 66 up to E12 and doubles daily from E12.5 to E14.5. This number then decreases beginning at E15.5[4]–[5], with HSCs finally fading away at 2–4 day postpartum (dpp)[6]. FL-HSCs are considered to subsequently seed the BM. In the second stage, hepatoblasts differentiate into hepatocytes and bile-duct epithelial cells, an event indicating the switch of the liver from a predominantly hematopoietic organ to a metabolic one. However, the molecular mechanisms underlying liver development both during and after hematopoiesis remain largely unknown.

The functional features of hematopoiesis and phenotype of the hematopoietic microenvironment are distinct in the FL compared to the BM or YS[7]. FL-HSCs have a much higher proliferative capacity for long-term repopulation than HSCs from BM or umbilical cord blood and acquire adult characteristics in mouse BM between 1 and 2 weeks after birth[8]–[9]. Co-culture of YS HSCs with FL nonhematopoietic cells has been shown to impart a long-term repopulating capacity to YS HSCs engrafting the BM[10]. Thus, the FL microenvironment not only stimulates expansion of the hematopoietic system, but also may modify the characteristics of HSCs. The FL stroma also behaves much more efficiently than the BM stroma to support hematopoiesis or differentiation of embryonic stem cells (ESCs) into hematopoietic cells[11]. However, the exact mechanisms that endow the FL microenvironment and FL-HSCs such specificities remain elusive.

Here, we used transcriptomic and proteomic approaches to analyze global changes in FL hematopoiesis in murine liver at E11.5, E14.5, E15.5, and 3 dpp. Our data not only profile the features and orchestration of transitional hematopoiesis and hepatogenesis, but also reveal that hematopoiesis emigration gates liver maturation.

## Materials and Methods

### Mouse strains

C57BL/6J mouse strains were bred and maintained at the National Institute of Biological Sciences (NIBS), Beijing. Age-matched embryos were generated by timed mating; the day of vaginal plug detection was considered to be E0.5. All animal experiments in this study were approved by the Institutional Animal Care and Use Committee (IACUC) of NIBS.

### Liver tissue collection

Twelve liver samples were collected from each of the five mice sacrificed at E11.5, E14.5, E15.5, and 3 ddp. Pregnant mice were killed by either cervical dislocation or decapitation. The uterus was removed and washed to remove maternal blood. Fetuses were dissected under a dissection microscope (SMZ1500; Nikon, Tokyo, Japan). The abdominal cavity was opened, and the liver under the septum transversum was removed, taking care to avoid removal of any adjacent tissues. The liver was shaken in ice-cold Hank's buffered saline solution (HBSS) to remove blood. A portion of the liver tissue was stored in RNAlater (Ambion, Austin, TX) for RNA extraction, while the remaining portion was directly frozen in liquid nitrogen and stored at −80°C for protein analyses.

### Microarray analysis

Liver RNA was prepared in triplicate using the RNeasy Mini kit (Qiagen, Valencia, CA). Poly(A)+ mRNA was purified from total RNA using the Oligotex Direct mRNA Midi/Maxi kit (Qiagen, Valencia, CA). The integrity of each RNA sample was assessed using an Agilent 2100 Bioanalyzer (Agilent, Palo Alto, CA). Double-stranded complementary DNA (cDNA) and labeled complementary RNA (cRNA) were synthesized from total RNA and hybridized, in replicate, to Affymetrix mouse 430 2.0 gene chips (Affymetrix, Santa Clara, CA). The chips were then processed and scanned with the GeneChip Scanner 3000. The resulting data were checked using GeneChip Operating Software (GCOS) v1.1.1 (Affymetrix).

### Differential in-gel fluorescence electrophoresis (2D-DIGE)

Liver tissue lysates obtained from animals at each time point (E11.5, E14.5, E15.5, and 3 ddp) were separated using 2-D DIGE as detailed elsewhere (Supplemental materials S1, Table S5)[12]. The resulting DIGE images were analyzed using DeCyder v.5.02 software (GE Healthcare) according to the instructions provided in the Ettan DIGE User Manual. Images of each gel were grouped into ‘standard’, ‘E11.5’, ‘E14.5’, ‘E15.5’, ‘3 dpp’. A one-way ANOVA was then used to compare each time point. Statistically significant spots (*P*<0.05) with an average volume ratio exceeding 2-fold were accepted for further identification.

### Protein identification with mass spectrometry (MALDI-TOF/TOF)

Protein spots of interest, as defined by the 2-D DIGE/DeCyder analysis, were excised from cCBB-stained gels for in-gel tryptic digestion and identified by MALDI-TOF-MS/MS as described previously[12]. The MS and MS/MS spectra were searched against the International Protein Index (IPI) mouse database version 3.18 (http://www.ebi.ac.uk/IP/IPIhelp.html) using GPS Explorer™ Version 3.0 and MASCOT 2.0[12]. All the proteins identified had protein scores greater than 59 (*P*<0.05) and individual ions scores greater than 21 (*P*<0.05). All the MS/MS spectra were further validated manually.

### Data analysis

Supervised hierarchical clustering of expression data was performed with the Cluster program 3.0 using Pearson correlation as similarity metric and centroid linkage clustering [13]. Clustering was visualized using the TreeView program[13]. Tissue expression profiles were generated using GeneAtlas, a gene expression database (http://symatlas.gnf.org/SymAtlas/) that contains gene expression patterns on a genome scale, as determined by tissue expression microarray on 79 human and 61 mouse tissues[14]. Functional annotation of genes and proteins was performed with the Protein ANalysis Through Evolutionary Relationships (PANTHER) program (http://www.pantherdb.org), which is based on Gene Ontology (www.geneontology.org/). Pathway analysis was performed using Ingenuity Pathway Analysis (IPA) (www.ingenuity.com), which is a robust analysis system and database (Ingenuity Pathways Knowledge Base, IPKB) that can be used to identify biological networks based on information extracted from peer-reviewed literature[15].

### Western blot analysis

Samples were separated on SDS-polyacrylamide gels and transferred onto nitrocellulose membranes in a trans-blot electrophoresis transfer cell (Bio-Rad, Hercules, CA). Membranes were blocked with 5% skim milk for 1 h, incubated with primary antibody (antibodies listed in Supplemental materials S1) at room temperature for 2 h, and then incubation with horseradish peroxidase-conjugated secondary antibody for 1 h at room temperature. The immunoreactive proteins were visualized with ECL reagents. All the membranes were exposed on the same X-ray film. A semi-quantitative analysis based on optical density was performed using QuantitiOne software (Bio-Rad, Hercules, CA).

### Semi-quantitative RT-PCR

Total RNA was harvested from mouse liver samples as described for microarray RNA isolation. Reverse transcription (RT) was performed with M-MLV RTase (Promega, WI, USA) and oligo dT(18). A fraction (1/25th) of the resulting RT product was subjected to 30 cycles of standard PCR, with each cycle consisting of 94°C for 30 s, annealing for 30 s, and 72°C for 30 s. Other conditions are provided in Table S6.

## Results

### Dynamic maps of mRNA and protein expression during liver development

To gain a better understanding the biological events occurring in the hematopoietic liver, we examined the liver transcriptomes and proteomes at four time points that correspond to the initiation (E11.5), peak (E14.5), recession (E15.5), and disappearance (3 dpp) of FL hematopoiesis[4], [6]. Quantitative proteomics was performed using 2-D DIGE. Differentially expressed protein spots, with abundance changes that were significant (*P*<0.05) and exceeded 2.0-fold, were filtered by performing comparisons between the livers at every two developmental stages (Figure S1; Table S1). The resulting protein spots were identified with MALDI-TOF/TOF. We successfully characterized proteins from 328 spots, representing 187 unique proteins (Table S2, S3). At the same time, expression arrays (Affymetrix mouse 420 2.0) identified 10,374 genes (partially shown in Table S4) expressed in the same liver samples mentioned above. A comparison of proteomic and transcriptomic data is shown in Supplemental materials S1 and Figure S5.

**Figure 1:**
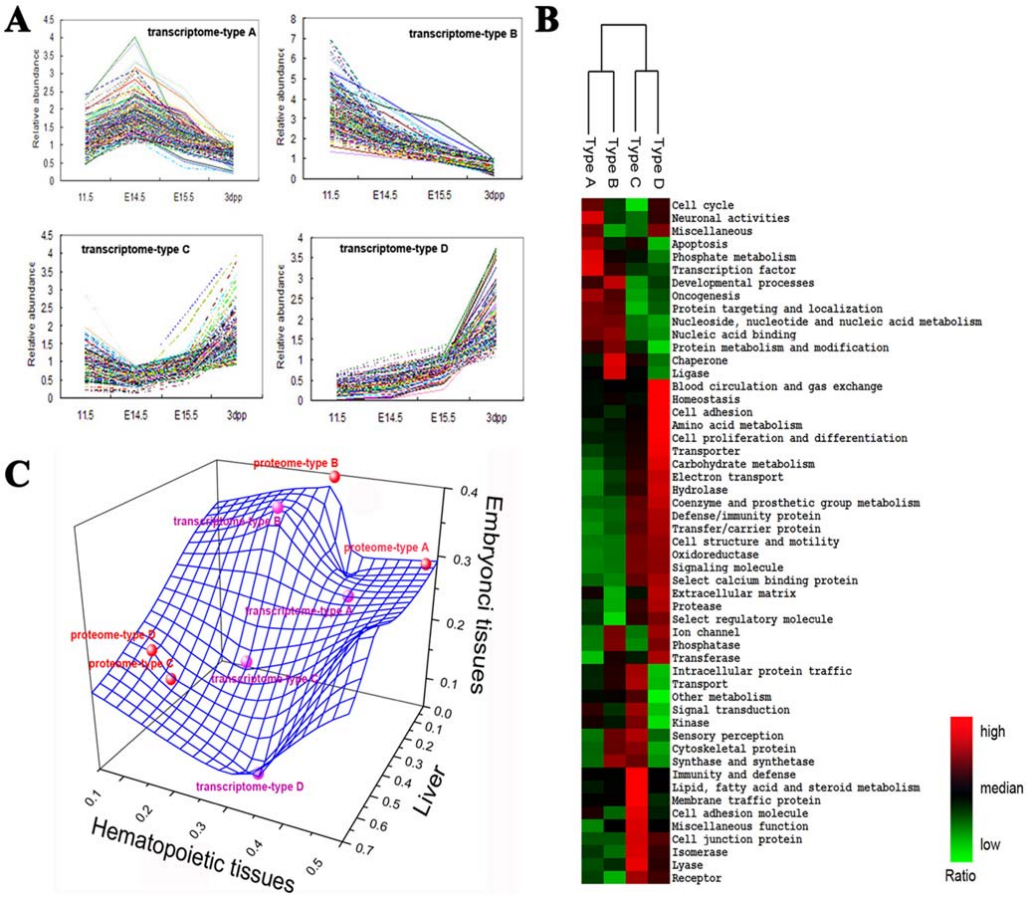
Development-associated changes in tissue expression patterns and the biological functions of the four dynamic types of liver genes. (A) The four dynamic types of liver genes with differentially expressed mRNA during E11.5, E14.5, E15.5, and 3 dpp. All the 1,428 differentially expressed genes exhibited mRNA changes between any two developmental stages that exceeded 2 fold. (B) Hierarchical clustering of biological functions for the four dynamic types. The biological functions are annotated by PANTHER. The ratio of each function in a type is expressed relative to its median ratio across all the four types and is depicted according to a color scale shown at the bottom. Red and green indicate the ratio of each function above and below the median, respectively. The magnitude of deviation from the median is represented by the color saturation. (C) Tissue expression patterns of the four dynamic types, shown for both the transcriptome and proteome. The three axes represent the ratio of the tissue-enriched genes in liver, hematopoietic tissue, and embryonic tissue. Gene tissue distribution information was annotated by GeneAtlas.

The FL genes could be clustered into four main types based on their distinct temporal expression profiles (types A–D, Figure 1A). Type A peaked at E14.5 and then faded from E15.5 to 3 dpp. Type B steadily decreased from E11.5 to 3dpp (data analysis shown in Supplemental materials S1). Type C was globally down-regulated around E14.5 and up-regulated thereafter. Type D progressively increased from E11.5 to 3 dpp. Functional clustering revealed that each of these four types of genes had different functional profiles (Figure 1B), which are described in the following section.

#### Tissue expression patterns

GeneAtlas analysis of both the transcriptome and proteome revealed that type A was dominated by hematopoietic tissue-enriched genes (48% of type A genes), type B with embryonic tissue-enriched genes (39% of type B genes), and type C and D with liver-enriched genes (51% and 61% of type C and D genes, respectively). In addition, type C and D genes extensively overlapped, implying that they may share similar biological functions (Figure 1C).

#### FL hematopoiesis— type A genes

Of the type A genes related to nucleoside, nucleotide, and nucleic acid metabolism, 60% were involved in mRNA transcription. In contrast, only 31% of type B genes had this function. Notably, 46% of the type A transcription factors were enriched in hematopoietic tissues; and 24% of those transcription factors enriched within hematopoietic tissues did not have a functional annotation. Approximately 50% of the type A transcription factors were KRAB box-containing zinc finger proteins (KRAB-ZNFs). KRAB-ZNFs were over two times more abundant in the type A group than in the type B group, though the total number of type B genes was double the number of type A genes. The type A group contained not only transcription factors, but also gene products involved in neuronal communication, such as adrenergic receptor β2 and opioid receptor κ1 (Figure 1B). Both receptors are known to affect BM hematopoiesis[16]. At the proteome level, the type A group was also abundant in functional groups involved in nucleoside, nucleotide, and nucleic acid metabolism as well as cell cycle, signal transduction, and protein metabolism and modification (Figure S2).

Type A genes were further matched to the Stem Cell Database (http://stemcell.princeton.edu)[17] to reveal any specificity to the characteristic properties of FL hematopoiesis. We found that type A genes had biological functions that were similar to those of FL-HSC-specific genes, but not those of LT-HSC-adult (BM)-specific genes (Figure S3). Shared functions included transcription, protein modification and metabolism, signal transduction, cell cycle, and development. As with type A transcription factors, which were predominantly (one-half) KRAB-ZNFs, one-half of FL-HSC-specific transcription factors were KRAB-ZNFs, and one-third of LT-HSCs transcription factors were KRAB-ZNFs.

**Figure 2:**
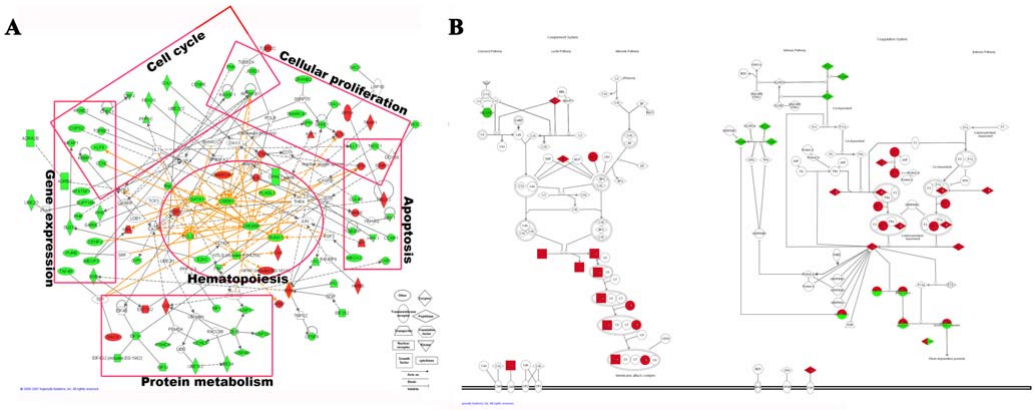
Pathway analysis of the dynamic types. (A) Type A network, developed by Ingenuity Pathway Analysis (www.ingenuity.com), integrating the transcriptome (green) and proteome (red). When a gene is shared by both transcriptome and proteome, we choose its data of proteome. The biological functions of the network are indicated with rectangles and ellipses. (B) Type C (green) and type D (red) pathways of blood coagulation and complement cascades. Pathways shared by both types are shown as half red and green (A symbol in the pathways represents a group of genes but not only one gene. Different Type C or D gene presents at the same symbol.).

Pathway analysis identified several distinct pathways that may participate in hematopoiesis. Combining transcriptomic and proteomic data generated an interaction network with specialized functions in hematopoiesis, cell cycle, cellular proliferation, gene expression, apoptosis, and protein metabolism (especially proteolysis) (Figure 2A). Factors known to be important for regulation of hematopoiesis as well as the commitment and self-renewal of HSCs formed the “central nodes.” These included CREBBP, RUNX1, NPM1, 14-3-3E, SMARCA5, BCL2L1, CREB, KITLG, and EZH2 (Figure 2A)[18]–[22]. However, proteolysis functions were not restricted to the type A group, as different components of the protein-ubiquitination pathway were represented in both type A and B (Table S4; Figure S4). Furthermore, 5 of the 11 type A ubiquitination-related molecules (PSMC6, USP14, UBE1L2, UBE2V2, and UBE2O) also appeared at the hematopoiesis peak in human FL (embryonic week 22, E22w), as we have previously shown by proteomic profiling[23]. As type B genes centered on embryonic development (Supplemental materials S1), distinct protein-ubiquitination pathways may be operative during FL-hematopoiesis.

#### Developmental patterns of liver gene expression during hematopoietic expansion— type C and D genes

Type C and D genes were primarily involved in liver-associated functions such as metabolism of lipids, fatty acids, steroids, carbohydrates, and amino acids. They were also involved in immunity and defense, cell structure and motility, signal transduction, transport, and homeostasis. Type C and D genes showed subtly different expression patterns. The former decreased during the expansion phase of FL hematopoiesis (from E11.5 to E14.5), while the latter increased continuously.

Type C mainly contained genes encoding mitochondrial, plasma membrane, and nuclear proteins involved in lipid and fatty acid metabolism, signal transduction, cell motility, and transport. This group also included genes involved in immunity and defense as well as macrophage- and NK cell-mediated immunity. Signal transduction genes made up 20% of type C genes and spanned over 55 pathways, including cell surface receptor-mediated signal transduction pathways and cell adhesion-mediated signaling pathways. Type D contained a high number of proteins involved in steroid metabolism. Half of type D genes resided at the extracellular space and plasma membrane.

The individual nature of type C and D genes was also apparent from analysis of networks. Type C genes were mainly involved in multiple aspects of carbohydrate metabolism, particularly carbohydrate utilization and catabolism. Functions of these genes included carbohydrate degradation, binding, conversion, and transport. On the other hand, type D genes were primarily involved in the production of carbohydrates or glycans, with the latter category making up 40% of carbohydrate metabolism genes of this type. Type C was unique in that it contained factors involved in energy production (APOM, ECH1, ENPP1, GBE1, CDO1, DHRS4, GRHPR, NNT, and PDIA5). Type C was also represented by gene products involved in the degradation of valine, leucine, and isoleucine as well as in acyl-CoA metabolism, fatty acid beta-oxidation, and metabolism of ketone bodies (SAT, PTGS1, ACOX1, HMGCS2). Type D stood out from type C in xenobiotic, steroid, and tryptophan metabolism with the presence of molecules such as cytochrome P450 enzyme, UDP-glucuronosyltransferase (UGT), multidrug resistance 1 (MDR1), aryl hydrocarbon receptor (AHR), and glutathione-S-transferase (GST). In the type D groups, a strong correlation existed between transcriptome and proteome for both glyconeogenesis (r = 0.676, *P*<0.05) and xenobiotic metabolism (r = 0.683, *P*<0.05). Type C and D were further represented by processes involved in blood coagulation and complement cascades, one of the main functions of the adult liver. Factors involved in intrinsic blood coagulation and classical complement cascades were clustered in type C, while those involved in the extrinsic blood coagulation and lectin and alternate complement cascades coagulation were classified as type D (Figure 2B). Both types shared genes involved in downstream pathways. With regard to hepatic system development, the type C group was rich in factors involved in liver proliferation and regeneration (ACOX1, PTGS1, and SAT). Type D factors participated in liver proliferation and size control of hepatocytes (TF, C5, and AHR).

At the proteomic level, factors involved in amino acid metabolism were abundant in the type C group, and protein levels were highly correlated with corresponding mRNA levels (r = 0.759, *P* = 0.0005). Likewise, a good correlation was present between proteomics and transcriptomics data for type C factors involved in carbohydrate metabolism (r = 0.49, *P* = 0.028) and HMGCS2 (r = 0.995, *P* = 0.005), the rate-limiting enzyme for ketogenesis. A correlation between proteomic and transcriptomic data was also present for type D factors involved in amino acid metabolism and energy metabolism (r = 0.45 and 0.429, respectively, *P*<0.01).

### E14.5-E15.5 is a key time point in liver development signaling the loss of hematopoiesis

**Figure 3:**
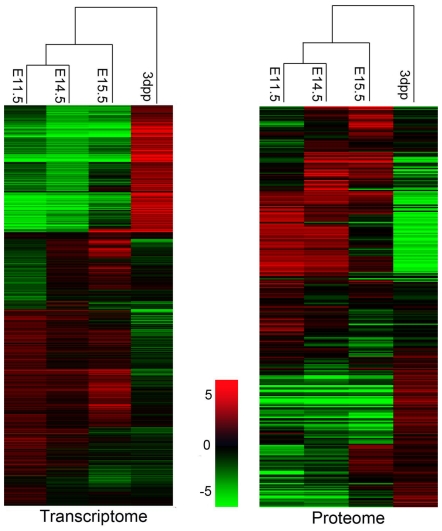
Cluster analysis showing mRNA and protein abundance for genes differentially expressed in mouse liver at E11.5, E14.5, E15.5, and 3 dpp. All the 2,593 differentially expressed mRNAs (left panel) and 187 differentially expressed proteins (right panel) changed more than 2-fold between any two developmental stages, as analyzed by Cluster 3.0 and TreeView. The expression level of each mRNA or protein at a development stage is shown relative to its median abundance across all the developmental stages and is depicted according to the color scale. Red and green indicate expression levels above and below the median, respectively. The magnitude of deviation from the median is indicated by color saturation.

The cluster analysis unexpectedly revealed that, at both a proteomic and transcriptomic level, highly significant differences existed between E14.5 and E15.5 compared to differences between E11.5 and E14.5 (Figure 3). This strongly suggests that E14.5 to E15.5 is a key time for liver development, with major changes occurring in mRNA and protein constituents and thus, the overall physiological processes. To gain better insight into the changes during this 24-h period, we focused on the mRNAs that changed by more than 2 fold and the proteins that changed by more than 1.5 fold. It is interesting that only 8% of these genes were shared by E11.5-E14.5 and E14.5-E15.5, pointing to differences in the developmental program at these times. PANTHER and IPA were applied in further functional analysis.

**Figure 4:**
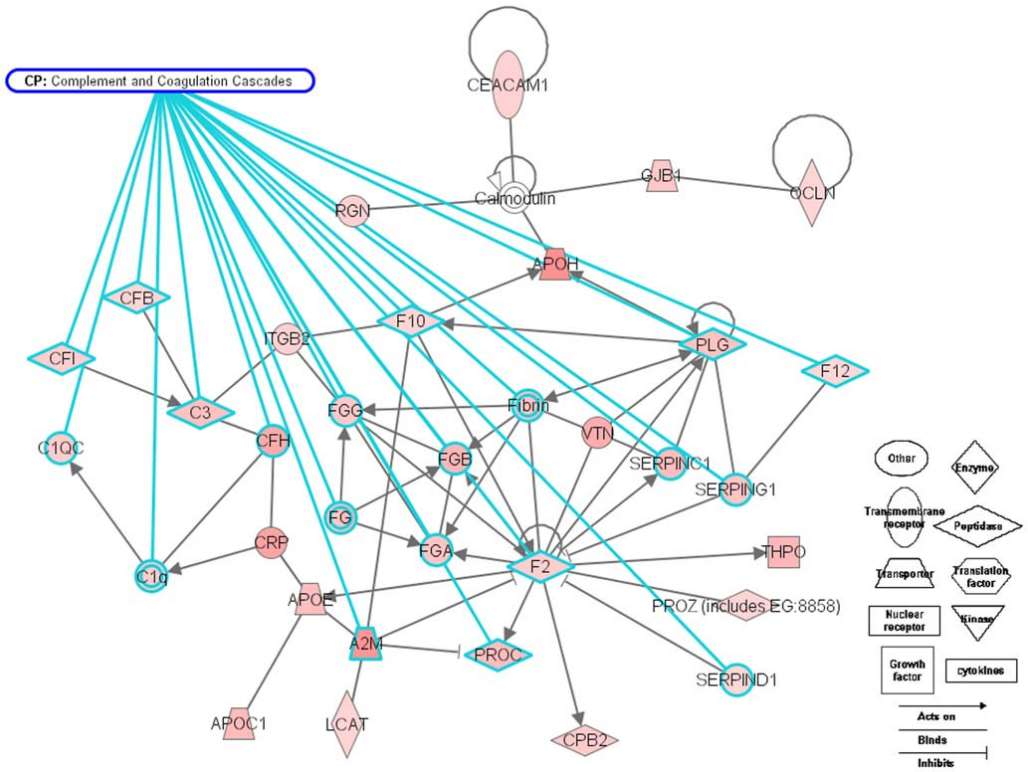
Up-regulation of blood coagulation and complement cascades at E14.5-E15.5. Genes that were related to blood coagulation and complement cascades and exhibited 2-fold up-regulation between E14.5 and E15.5 were filtered and integrated into a network using Ingenuity Pathway Analysis (www.ingenuity.com). Genes related to blood coagulation and complement cascades are tethered to the label with green lines. The saturation of red shading indicates the degree of up-regulation.

Suppression of hematopoiesis was associated with down-regulation of factors such as GATA4, VEGFC, SMARCA4, CDK6, and MYC. It was also associated with up-regulation of factors such as LCN2, DDIT3, and RNASEL, which would be expected to enhance hematopoietic cell apoptosis. Similar changes occurred at the proteomic level (i.e., the hematopoiesis-associated proteins, NPM1, EEF2 decreased). At the same time, the migration of hematopoietic cells would be reduced by the increased expression of a number of migration inhibitors in the type C group. Of the total 117 up-regulated network-eligible genes, 48 participate in blood coagulation and cell movement in hematological system development and function. The expression of both proteases and protease inhibitors, including many serine proteases and serine protease inhibitors, was increased (Figure 4). This was one of the changes signaling that the newly developed liver was fully competent to fulfill its functions.

The major biological processes of the adult liver (i.e., immunity and defense, metabolism of materials) were increased, as reflected by changes in both the transcriptome and proteome. Various transferases, oxidoreductases, and lyases were significantly up-regulated, indicating an increase in the metabolism of lipids, carbohydrates, proteins, and amino acids as well as the transport of electrons, lipids, and fatty acids. The main xenobiotic metabolic pathways, including those involving nuclear factor E2-related factor 2 (Nrf2), constitutive androstane receptor (CAR), pregnane X receptor (PXR), and aryl hydrocarbon receptor (AHR), were up-regulated with the increased expression of the enzymes UGT, GST, FMO, ALDH, and CYPs. Proteins that are known to participate in liver development, including C3, C5, and IGFBP-1, were up-regulated. MYC, CCND1, and GH1 were down-regulated, controlling the size and proliferation of the liver.

### E11.5 to E14.5 is the hematopoietic expansion phase and is associated with depression of some liver functions

As already mentioned, analysis of dynamic maps revealed that, during E11.5 to E14.5, genes involved in FL hematopoiesis and hepatogenesis were differentially regulated. To confirm whether these were significant events, we focused on genes undergoing at least a 2-fold change in expression and proteins undergoing a 1.5-fold change. Geness involved in hematopoiesis or proliferation of hematopoietic cells, particularly those required for maintaining HSCs self-renewal, were increased in parallel with increased expression of activators of ITGA4, RUNX1T1, CCR2, CD24 and CXCL3 and with decreased expression of suppressors of TGF-β1, SMO, ID1, ID2, PRTN3 and E2A, and THPO. It was apparent that the decreased HSC generation triggered by C-MYC was important to maintain a homeostatic balance between hematopoiesis and hepatogenesis. Accordingly, markers of erythropoiesis, hemoglobin alpha and beta, and uroporphyrinogen decarboxylase were up-regulated. KITLG and ITGA4, putative regulators of HSCs motility, increased significantly.

Striking decreases occurred in glycosyltransferases, carbohydrate kinases, actin-binding cytoskeletal proteins, ribosomal proteins, and translation factors. This suggests that, from E11.5 to E14.5, carbohydrate and amino acid metabolism, protein biosynthesis, general vesicle transport, cell structure changes, cell motility, DNA repair, and DNA replication were considerably suppressed. CPT1A (carnitine palmitoyltransferase 1a), a liver-specific gene that is the rate-limiting enzyme for the fatty acid oxidation pathway, was down-regulated 2.5 fold. Some forms of lipid metabolism, steroid metabolism, and lipid and fatty acid transport were elevated; however, fatty acid metabolism was inhibited.

### Validation of protein and mRNA expression by western blot and RT-PCR

**Figure 5:**
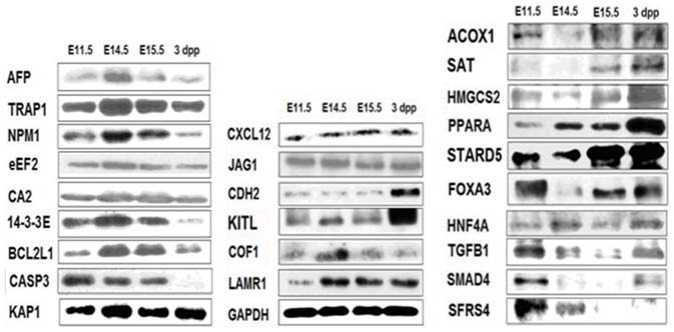
Validation of proteomic changes by western blot. Expression of select proteins in the mouse liver at E11.5, E14.5, E15.5, and 3 dpp. Proteins that were analyzed included those exhibiting a type A pattern of expression (left column), those related to the mobilization of hematopoietic stem cells (middle column), and those related to liver function or other functions (right column). GAPDH served as an internal control.

**Figure 6:**
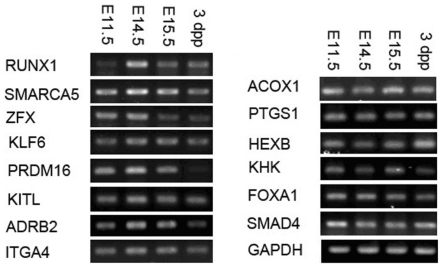
Validation of transcriptomic changes by RT-PCR. The mRNA expression of select mouse liver genes at E11.5, E14.5, E15.5, and 3 dpp. Genes that were analyzed included genes related to hematopoiesis (left column) and genes related to liver function and development (right column). GAPDH served as an internal control.

Changes in molecules associated with hematopoiesis, HSC motility, liver development, and TGF-β signaling were confirmed by western blot and RT-PCR (Figures 5–6). For all factors, the results were consistent with observations obtained through proteomic or transcriptomic approaches. Type A factors associated with FL hematopoiesis-related factors (such as AFP, TRAP1, NPM1, eEF2, CA2, 14-3-3E, BCL2L1, RUNX1, and SMARCA5) increased to E14.5 and decreased thereafter as seen by western blot and RT-PCR (Figures 5–6). KRAB-ZFPs, ZFX, KLF6, and PRDM16 as well as their specific KRAB-binding protein, KAP1 (KRAB-Associated Protein 1)[24] were expressed in the same manner (Figure 5). To determine if the putative molecules controlling cell movement in BM may also function in the movement of FL hematopoietic cells, we analyzed CXCL12, JAG1, and CDH2 expression (Figure 5). In contrast to KITL, COF1, LAMR1, ADRB2, and ITGA4 (Figures 5–6), these proteins did not change during FL hematopoiesis.

Type C-like expression profiles of other important enzymes and proteins participating in liver metabolism were also validated by western blot and RT-PCR (Figures 5–6). These included ACOX1, SAT, PTGS1, HEXB, KHK, HMGCS2, STARD5, and the related transcription factors PPARA, FOXA3 (HNF3G), FOXA1 (HNF3B), and HNF4A. Finally, validations were performed for TGF-β and SMAD4 (Figures 5–6), TGF-β signaling pathway components that, aside from acting as inhibitors of hematopoiesis, also inhibit hepatocyte proliferation during the development of late fetal to postnatal liver[3].

## Discussion

FL-HSCs undergo a high frequency of cycling and self-renewal until E14.5, contrasting sharply to the quiescent and limited self-renewal of BM-HSCs[4].This suggests that the FL provides a microenvironment that is more conducive to supporting HSCs. For this reason, FL is considered a good model to study the molecular partners governing the self-renewal and proliferation of HSCs. In this report, we describe, for the first time, a global view of the development of FL hematopoiesis at the molecular level. Genes fluctuating strictly in accord with the development of FL hematopoiesis (type A) were primarily involved in hematopoietic expansion and were FL-HSC specific. Hepatic progenitors probably contributed to the development of FL hematopoiesis, with some pathways being restrained, especially metabolic pathways producing reactive oxygen species (ROS).

Compelling evidence supports the idea that intrinsic and extrinsic cellular mechanisms synergistically limit the expansion and differentiation of stem cells, especially HSCs[25]. Here, integration of transcriptomic and proteomic data identified transcription factors that fluctuated during FL hematopoiesis (type A) and were knitted into an interaction network (Figure 2A). Some of these transcription factors are known to control the self-renewal of HSCs or hematopoietic commitment of ESCs. BCL2L1, NPM1, SMARCA5, EZH2, KITLG, and CREBBP (CREB-binding protein) contribute to the formation, self-renewal, long-term survival, or complete long-term repopulating potential of the hematopoietic stem cell population[18]–[22]. As the central “nodes” in the network (Figure 2A), these factors control the expression of other partners, as reflected in both the proteome and transcriptome. GATA1 and BCL2L1, which exhibit a type A pattern of transcription, interact with the type A proteins HBA2 and TPT1, respectively[26]. At E11.5-E14.5, the hematopoiesis inhibitors were significantly suppressed. These included TGF-β signaling components (TGF-β1, TGF-β2, SMAD4, and SMAD7), a Hedgehog signaling protein (SMO), and IDs (DNA-binding protein inhibitor, ID) (ID1 and ID2) [27]–[29]. It is important to note that this protein profile is probably distinctive for FL hematopoiesis, as suggested by a comparison of FL-HSC- and LT-HSC-BM-specific genes (Figure S3), and many type A genes were newly identified as important for hematopoiesis or as having no clear functional annotation. The results relating to type A factors, as well as other dynamic types, demonstrate that the experimental approaches we used were successful for characterizing the features of FL hematopoiesis and other type-specific biological functions.

Many type A transcription factors identified here had unknown functions. Half of these were hematopoietic tissues-enriched and over half were KRAB-ZNFs, agreeing well with proteomic data obtained in human FL at the hematopoietic peak[23]. In recent years, more and more KRAB-ZNFs have been shown to be influential to hematopoiesis regulation. Among the KRAB-ZNFs identified here, ZFX, KLF6, and PRDM16 are already known to be important hematopoietic regulators. For instance, ZFX regulates the self-renewal of ESCs and HSCs[30]. We found that KAP1, a specific, high-affinity KRAB-binding protein[24], had an expression pattern similar to that of KRAB-ZFPs, inferring that KAP1 may assist KRAB-ZFPs in promoting FL hematopoiesis (Figure 5). Taken together, these results also suggest that the transcription factors mentioned control the self-renewal and proliferation of HSCs.

If transcriptional regulation is an intrinsic element governing FL hematopoiesis, then the FL hematopoietic microenvironment is an extrinsic one. Given their direct spatial relationship with hematopoietic cells, hepatic progenitors are the most likely cell type that assembles the FL hematopoietic microenvironment in concert with other stromal cell populations. Hepatic cells or nonhematopoietic cells isolated from FL at E14.5 or E15 support proliferation of hematopoietic progenitors from FL and adult BM in co-culture; however, this is not the case with cells obtained at E18.5[10], [31]. Obviously, hepatic cells at the hematopoietic expansion phase are very different from those at the hematopoietic shrinking phase.

Our data revealed that liver functions were partially repressed during the FL hematopoietic expansion phase (from E11.5 to E14.5) but were significantly increased thereafter (at E15.5, type C genes). These liver functions included metabolism of lipids, carbohydrates, and amino acids, in particular catabolism/utilization of materials and energy production processes such as fatty acid beta-oxidation, ketogenesis, and degradation of carbohydrates and amino acids. Other liver functions were also increased, as seen by up-regulation of factors involved in classical complement cascades and intrinsic coagulation pathways. An example of a change associated with increased metabolic liver functions at E15.5 is up-regulation of HEXB, which hydrolyzes terminal non-reducing N-acetyl-D-hexosamine residues into N-acetyl-beta-D-hexosaminides. We also observed an up-regulation of KHK (ketohexokinase or fructokinase), which initiates the intracellular catabolism of a large proportion of dietary carbohydrates, as well as an up-regulation of STARD5, a cholesterol-binding protein that does not have well-understood functions but is thought to be important for cholesterol metabolism and maintenance of cellular homeostasis[32].

Surprisingly, liver functions emerging at E15.5 included not only those functions that were suppressed during hematopoietic expansion, but also functions that continued to increase throughout liver development. These functions included biotransformation, steroid and tryptophan metabolism, extrinsic blood coagulation, and the lectin pathway of complement cascades. This intimates that the development of the FL is delayed with undifferentiated hepatic progenitors during the FL hematopoietic expansion phase and then enters a full-scale maturation stage as soon as FL hematopoiesis begins receding. The delay in liver development is probably required for FL hematopoiesis, as evidenced by the co-culture of primitive hepatocytes with HSCs. FL stromal cell populations at E12.5 to E14.5 with epithelial-to-mesenchymal transition features (a characteristic of hepatoblasts) have hematopoietic supportive capacity only before oncostatin M-induced hepatocytic differentiation[33]. Moreover, Dlk, a specific marker of hepatic progenitors, seems to regulate the hematopoietic-supporting capacity of liver stromal cells[34]. Therefore, hepatic progenitors are an important component of the FL microenvironment at this time point. Indeed, liver parenchyma is enriched in hepatoblasts during the expansion period of FL hematopoiesis. E14 rat liver possesses a higher proportion of hepatoblasts than the E18 liver[35]. Mouse FL also expresses higher levels of Dlk1 during E12.5-E16.5 than during later phases[36]. Type C genes probably reflect the remarkable nature of hepatic progenitors in the FL.

Multiple mechanisms may account for the ability of hepatic cells to contribute to the FL hematopoietic microenvironment during the hematopoietic expansion phase. First, hepatic cell-derived cytokines that negatively regulate hematopoiesis seem to be limited when FL-HSCs are expanding. Liver is the primary organ of thrombopoietin production, and hepatocyte is consider to be the only producer of this cytokine in fetal liver[37]. Thrombopoietin/MPL (thrombopoietin receptor) signaling is crucial for maintaining HSC quiescence in the postnatal hematopoietic niche, and decreases in thrombopoietin do not inhibit development of fetal HSCs in thrombopioetin knockout mice[38]. The 2.8-fold decrease in thrombopoietin (a type C factor) observed during E11.5-E14.5 suggests that the temporal decrease in thrombopoietin in hepatocytes is required to support the long-term self renewal and proliferation of HSCs during the expansion phase of FL hematopoiesis. An extra benefit is that decreased thrombopoietin reduces ROS generation[39]. Second, we found some ROS generation pathways in fetal liver are limited to FL hematopoiesis. A hypoxic milieu is essential for the survival of HSCs and stromal cells and provides long-term protection from oxidative stress, as occurs in FL hematopoiesis[40]. A hypoxic microenvironment contributes to increases in the hematopoietic regulators mentioned above, such as CREBBP1, NPM1, KITLG, and BCL2L1[41]–[42]. Amazingly, we found that some rate-limiting enzymes in ROS generation pathways were down-regulated during the expansion phase of FL hematopoiesis and then up-regulated as FL hematopoiesis began to come to an end (type C factors). Among those factors, changes in three ROS-producing factors (ACOX, PTGS1, and SAT) were further confirmed at the mRNA or protein level (Figures 5–6). ACOX1 (acyl-coenzyme A oxidase) is the rate-limiting enzyme for the peroxisomal very-long-chain fatty acid beta-oxidation pathway, which controls the generation of hydrogen peroxide. Overexpression or deficiency of ACOX1 will increase ROS and hepatocyte proliferation[43]. PTGS1 (prostaglandin-endoperoxide synthase), also known as COX-1 (cyclooxygenase-1), is the rate-limiting enzyme for the pathway that catalyzes the regio- and stereo-specific oxygenation of polyunsaturated fatty acids to produce prostaglandin, thromboxane, and hydroperoxide products. Inhibition of PTGS1 alone tends to delay liver regeneration[44]. SAT (spermidine/spermine N1-acetyltransferase) is the only rate-limiting enzyme for polyamine degradation, which generates hydrogen peroxide as a byproduct. Increased SAT levels deplete hepatic polyamine levels, making these levels insufficient for initiation of early liver regeneration[45]. Thus, all three of these factors not only produce ROS, but also control hepatocyte proliferation through ROS production.

The changes of those ROS pathways or other metabolic pathways might be ascribed to liver-enriched transcription factors. HNF4α (hepatocyte nuclear factor 4 alpha) and FOXA3 are two of the most important regulators of hepatogenesis and potential transcriptional controllers of metabolism-associated genes[46]–[48]. HNF4α, a repressor the vital enzymes of ROS pathways mentioned, decreased between E11.5 and E14.5, while it increased at E15.5. HNF4α might negatively regulate the expression of CPT1A (2.5-fold decrease from E11.5 to E14.5), HMGCS2, and ACOX1 as well as control hydrogen peroxide production in peroxisomal oxidation[47]–[48]. However, the exact mechanisms regulating these changes during FL hematopoiesis are unknown.

The up-regulation of xenobiotic metabolizing enzymes and antioxidant enzymes that was observed between E11.5 and E14.5 was probably related to the suppression of the genes mentioned above as well as their key transcription factor, PPARA (Figure 5). PPARA is not indispensable for transcriptional regulation of ACOX1; however, in mice with ACOX1 deficiency or in which peroxisomal fatty acid beta-oxidation has been disrupted, PPARA and its targeted genes are up-regulated[43]. Here, when ACOX1 down-regulated during the expansion phase of FL hematopoiesis, PPARA-regulated proteins (CYP4s, EHHADH, and FABP) were up-regulated (Table S2). Overexpression of PPARA elevates the activity of the antioxidant enzymes catalase and SOD[49]. We also found that protein levels of the antioxidant enzymes catalase, SOD, PRDX1, PRDX2, and glutathione transferases were up-regulated (Table S2).

Taken together, our results reveal that, during the hematopoietic expansion phase, the FL seems to have a full complement of hepatic progenitors, though only a limited development of liver functions occurs. This is especially true of those depressed functions, which are attributed to building hematopoietic microenvironment, such as the ROS-producing pathways. However, as soon as hematopoiesis begins to fade at E14.5-E15.5, all of FL functions are wholly promoted. In conclusion, under the selective pressure, liver development is partially limited to support hematopoiesis. And the removal of hematopoiesis opens the door of liver maturation. Thus, our research globally evidences that the shift of hematopoiesis at E14.5-E15.5 is a key time point at which the primary development of the FL shifts to a maturation stage. How these features of FL development are shaped by intrinsic machinery or hematopoietic stress is currently unknown and awaits further investigation.

## Supporting Information

Figure S1 The differentially expressed protein spots displayed by merged the gels of mice livers of E14.5 and 3 dpp. Green spots are highly expressed in E14.5, red ones are highly expressed in 3 dpp liver, and the white ones are expressed in the equal abundance in the both.
(1.80 MB EPS)

Figure S2 Hierarchical clustering of biological functions and four dynamic types based on protein expression data. The biological functions are annotated by PANTHER. The ratio level of each function in a type is relative to its median ratio across all the four types and is depicted according to a color scale shown at the bottom. Red and green indicate the ratio of each function of a type respectively above and below the median. The magnitude of deviation from the median is represented by the color saturation.
(2.32 MB EPS)

Figure S3 Comparison of the genes of type A with the mouse FL-HSCs specific, LT-HSC-adult (BM) specific genes from the Stem Cell Database (SCDb) constructed by Phillips R.L. et al [24]. The X-axis was the different biological functions, and the Y-axis was the ratio of the genes of each function to the total gene of the three compared gene groups (type A of our data, the mouse FL-HSCs specific, LT-HSC-adult (BM) specific genes).
(0.64 MB EPS)

Figure S4 Protein ubiquitination pathways in type A and B identified by Ingenuity Pathway Analysis (www.ingenuity.com). The red labeled nodes were from type A, and the green ones from type B, and the half red and green ones were shared by both types.
(1.98 MB EPS)

Figure S5 Comparison between the transcriptomes and proteomes. (A) The tissue-specific or -enriched expression profile of the proteins with or without significant relationship to their mRNA. Mouse gene expression information of embryonic tissues including blastocysts and embryonic tissues at E6.5-E10.5, and hematopoietic tissues including bone marrow, thymus, spleen, lymphoid and lymph cells were referenced from GeneAtlas (http://symatlas.gnf.org/SymAtlas/) annotation. (B) The correlation between transcriptome and proteome for individual biological function.
(1.24 MB EPS)

Supplemental Materials S1 Materials and methods; Comparison of quantitative liver transcriptomes and proteomes;Recession of the early embryonic development genes - type B.
(0.09 MB PDF)

Table S1 Differentially expressed protein spots for mouse livers of the four different developmental stages
(0.03 MB DOC)

Table S2 Proteome data
(0.41 MB XLS)

Table S3 Transcriptome data
(1.46 MB XLS)

Table S4 Components of protein-ubiquitination pathway emerged in type A and B
(0.03 MB DOC)

Table S5 Experimental design of DIGE. Mice livers of three stages, E11.5, E14.5, E15.5 and 3 dpp, For each sample pair, triplicate gels were run, which are represented by number 1, 2 and 3.
(0.03 MB DOC)

Table S6 PCR Primers and PCR Conditions
(0.06 MB DOC)
