# Supplementary material for: Relationships between Hematopoiesis and Hepatogenesis in the Midtrimester Fetal Liver Characterized by Dynamic Transcriptomic and Proteomic Profiles
Source: PLoS One. 2009 Oct 28;4(10):e7641. doi: 10.1371/journal.pone.0007641 (PMC2765071; doi:10.1371/journal.pone.0007641)
Supplement: Supplemental Materials S1 — Materials and methods; Comparison of quantitative liver transcriptomes and proteomes;Recession of the early embryonic development genes - type B. (0.09 MB PDF) [file pone.0007641.s006.pdf]

Supplement materials and methods:

Antibodies. anti-14-3-3E (Genway, San Diego, CA, USA), anti-ACOX1 (ProteinTech, Chicago, IL, USA), anti-AFP (Sigma, Saint Louis, MI, USA), anti-BCL2L1 (Abcam, Cambridge, UK), Anti-CA2 (Santa Cruz, Santa Cruz, CA, USA), anti-CASP3 (Cell signaling, Danvers, MA, USA), anti-CDH2 (Cell signaling, Danvers, MA, USA), anti-COF1 (Abcam, Cambridge, UK), anti-eEF2 (Cell signaling, Danvers, MA, USA), anti-FOXA3 (Santa Cruz, Santa Cruz, CA, USA), anti-HMGCS2 (Genway, San Diego, CA, USA), anti-HNF4A (Santa Cruz, Santa Cruz, CA, USA), anti-JAG1 (BD, San Jose, CA, USA), anti-KITL (Biovision, Mountain View, CA, USA), anti-KAP1 (gifted from Dr. Dong Yang, BPRC), anti-LAMR1 (ProteinTech, Chicago, IL, USA), anti-NPM (Cell signaling, Danvers, MA, USA), anti-PPARA (Abcam, Cambridge, UK), anti-SAT (ProteinTech, IL, USA), anti-SDF-1 (RD, Minneapolis, MN, USA), anti-SFRS4 (ProteinTech, Chicago, IL, USA), anti-SAMRD4 (Abcam, Cambridge, UK), anti-SRARD5 (ProteinTech, Chicago, IL, USA), anti-TGFB1 (Abcam, Cambridge, UK), anti-TRAP1 (BD, San Jose, CA, USA), anti-GAPDH (Kang Chen, Shanghai, China), anti-rabbit HRP, anti-Goat HRP, anti-chicken IgY HRP and anti-mouse HRP (Santa Cruz, Santa Cruz, CA, USA).

Sample Preparation for DIGE. Liver tissues were ground into fine powder in liquid nitrogen and homogenized in lysis buffer (7M urea, 2M thiourea, 4% CHAPS, 10mM Tris, 5mM magnesium acetate, one complete proteinase inhibitor cocktail tablet per 50ml lysis buffer). For improved cell lysis, the solution was sonicated on ice for 1min with 1 second pulse on and 1 second pulse off to prevent overheating. The samples were incubated for

30min at room temperature with repeated vortexing, and then centrifuged at 40,000g for 60min at 20℃. The supernatant was stored in aliquots at -80℃. Protein concentration was determined with the Bradford assay kit (Bio-Rad, Hercules, CA, USA) by using albumin diluted in lysis buffer as standard.

Two-dimensional DIGE. Liver tissue lysates were labeled with Cy2, Cy3, and Cy5 following the protocols described in the Ettan DIGE User Manual (18-1164-40 Edition AA, GE Healthcare, Little Chalfont Bucks, UK). The DIGE experimental design was shown in Table S5. Typically, fifty micrograms of lysates were labeled with 400 pmol of Cy3 or Cy5, while the same amount of the pool standard that contained the equal quantities of all the samples was labeled with Cy2. To achieve statistical confidence, each sample was reloaded on three gels. Labeling reactions were carried out on ice and in the dark for 30 minutes before being quenched with 1μl of 10 mM lysine for 10 minutes on ice. These labeled samples were then combined for 2-D DIGE analysis. Two-dimensional gel electrophoresis was performed as described earlier with some modifications(Zhang et al., 2006). IPG strips (24cm, pH3-10, and NL) were rehydrated with labeled samples in the dark overnight with rehydrated buffer (8 M urea, 4% w/v CHAPS, 20 mM DTT, and 1% (v/v) IPG buffer and trace amount of bromophenol blue). First-dimension IEF was performed using an Ettan IPGphor System (GE Healthcare) for a total of 87 kVh at 20°C. The strips were then treated with a two-step reduction and alkylation step prior to the second dimension (SDS-PAGE). After equilibration with a solution containing 6 M urea, 30% glycerol, 2% SDS, 50 mM Tris-HCl, pH 8.8 and 0.5% (w/v) DTT, the strips were treated with the same solution containing 4.5% (w/v) iodoacetamide instead of DTT. The strips

were over-layered onto 12% polyacrylamide gels (20 x 24 cm), immobilized to a low-fluorescent glass plate and electrophoresed for ~10 h at 30 mA per gel using an Ettan DALT Twelve System (GE Healthcare). The Cy2, Cy3, Cy5-labeled images were acquired on a Typhoon 9410 scanner (GE Healthcare).

Image analysis. DeCyder v.5.02 was used to analyze the DIGE images as described in the Ettan DIGE User Manual (GE Healthcare). Intra-gel spot detection, quantification and inter-gel matching and quantification were performed using Differential In-gel Analysis (DIA) and Biological Variation Analysis (BVA) modules respectively. Briefly, in DIA, the Cy2, Cy3 and Cy5 images for each gel were merged, spot boundaries were automatically detected, and normalized spot volumes (protein abundance) were calculated. The resulting spot maps were exported to BVA. Matching of the protein spots across 6 gels was performed after several rounds of extensive land marking and automatic matching. Dividing each Cy3 or Cy5 spot volume with the corresponding Cy2 (internal standard) spot volume within each gel gave a standard abundance, thereby correcting inter-gel variations. Each gel was first grouped into 'E11.5', 'E14.5', 'E15.5', '3 dpp'. To test for significant differences in expression of proteins between every two groups, one-way analysis of variance (ANOVA) was performed at a statistical significance level of 0.05. The differentially expressed protein spots were filtered based on an average volume ratio of 2-fold with statistical significance ($p<0.05$).

Peptides extracted for identification. Separate preparative gels were run to obtain sufficient amounts of protein for MS analysis. These gels were fixed and stained with colloidal CBB (cCBB). Protein spots of interest, as defined by the 2-D DIGE/DeCyder

analysis, were excised from the cCBB-stained gels for a modified in-gel tryptic digestion procedure. Gel pieces were first discolored in 50% acetonitrile and 25mM ammonium bicarbonate and then subjected to reduction and alkylation in 10 mM DTT and 55 mM iodoacetic acid respectively. Following vacuum drying, the gel pieces were incubated with sequencing grade modified trypsin (Promega, WI, USA) at a final concentration of 0.01µg/µl in 25mM ammonium bicarbonate for 16 h at 37 ºC. Supernatants were collected, vacuum-dried, redissolved in 50% ACN and 0.1% TFA for MS analysis.

MALDI TOF/TOF analysis. Peptides were mixed with MALDI matrix (7mg/ml CHCA and 0.1% TFA and 50% ACN) and spotted on to the 192-well stainless steel MALDI target plates. Samples on the MALDI target plates were then analyzed using an ABI 4800 Proteomics Analyzer MALDI TOF/TOF mass spectrometer (Applied Biosystems). For MS analyses, typically 1000 shots were accumulated for each spot, while for MS/MS analysis, 2500 shots were accumulated. MS/MS analyses were performed using air, at collision energy of 1 kV and a collision gas pressure of 2.0×10^{-8} to 3.0×10^{-7} Torr. The MS together with MS/MS spectra were searched against the International Protein Index (IPI) mouse database version 3.18 (http://www.ebi.ac.uk/IP/IPIhelp.html) using the software GPS Explorer™ Version 3.0 and MASCOT database search algorithms (version 2.0). Cysteine carbamidomethylation and methionine oxidation were selected as variable modifications. One missing cleavage was allowed. Precursor error tolerance was set to < 0.1 Da and MS/MS fragment error tolerance < 0.25 Da. All the proteins identified should have protein scores greater than 59 ($p<0.05$) and individual ions scores greater than 21 with expect value < 0.05. All the MS/MS spectra were further validated manually.

Results:

Comparison of quantitative liver transcriptomes and proteomes

When compared the correlation of the proteomic and transcriptomic data, 41 proteins were followed the same temporal profiles significantly with their mRNA, another two with negative relation($r>0.95$ or <-0.95 and $p<0.05$). This is similar to the correlationship between the temporal profiles of proteome and transcriptome in other reports(Chen et al., 2002; Yoon et al., 2003).

To reveal the physiological relevance of the alteration of gene expression and their relationship with the corresponding protein expression, we firstly clustered the genes based on the gene expression database GeneAtlas (http://symatlas.gnf.org/SymAtlas/). The genes that were expressed in a tissue more than 3 times of the medians of abundances across 61 mouse tissues were considered to be enriched. When considering genes that are enriched in liver, hematopoietic tissues (including bone marrow, umbilical cord, spleen, thymus, lymphonode and lymphocytes) or early embryonic tissues (including blastocysts and embryonic tissues between E6.5 and E10.5), it became apparent that their expression panels were quite different between the proteins with or without significant relationship to their mRNA (Fig. S5A). Thus, in the majority of cases (67%) there was a positive correlation between the alterations of liver-enriched protein expression to the corresponding changes of gene expression. This class of proteins underlies general liver functions, such as metabolisms of carbohydrate, amino acid, lipid, fatty acid and steroid etc.

This was also indicated in the correlation between transcriptome and proteome for

individual biological function (Fig. S5B). Proteins with strong correlation to gene expression corresponded to the main liver functions including blood coagulation, xenobiotic metabolism, protein synthesis and cholesterol metabolism ($r \geq 0.90$, $p<0.05$). The medium correlation groups included coenzyme metabolism, electron transport, amino acid metabolism, proteolysis, lipid metabolism and transcription ($r \geq 0.40$, $p<0.05$). Unexpectedly, some of the proteins involved in transport, antioxidation and free radical removal, carbohydrate metabolism and small molecule biochemistry showed a lower relation ($r<0.40$, $p<0.05$). Protein groups with no significant relationship ($p>0.05$) to their mRNA including immunity and defense, cell signaling, cell cycle, cell structure and motility, RNA post-transcriptional modification, protein modification, cellular growth and proliferation, stress response and cell death were also enriched in hematopoietic and early embryonic tissues in most cases (94%).

Recession of the early embryonic development genes - type B. Genes of type B showed stealthy decrease during liver development. Seventy percentages of the genes were involved in nucleoside, nucleotide and nucleic acid metabolism, protein metabolism and modification. These genes played important roles in transcription and mRNA splicing. Among transcription factors, about 30% clearly directed developmental processes, 47% of which expressed richly in early embryonic tissues, and 30% were transcription cofactors. Furthermore, type B genes covered more than 70% and 80% of methyltransferases and chromatin/chromatin-binding proteins, respectively, across all the 4 dynamic types of transcriptome, about half of which were early embryonic tissue-enriched. For instance, mRNA splicing factors, ligases and chaprones in charge of mRNA splicing and

post-translational modification were above 52%, 45% and 60% of those genes highly enriched in embryonic tissues, respectively.

One of type B process was grouped with early embryonic development. Consistent to PANTHER, gene expression, RNA processing and protein posttranslational modification were the main functions for networks of type B. Concretely, RNA splicing and protein folding were prevalent, implying that those were the main approaches to control the expression of some genes, especially, of those associated early embryonic development. The proteins in this class included SUMO1, VDAC1, PICH1, p12, DNMT1, EME1(Abraham et al., 2003), HMGA1, UHRF1 and POLR2C for proliferation and specification of embryonic stem cells (ES); MYH10 and TGFB2 for the formation of outflow tract; ARFRP1, LDB1, NSD1 and NUP98 for gastrulation. This was supported by the proteomic data of the same dynamic map with two splicing factor HNRPC and HNRPH1. Of genes related to DNA replication, recombination and modification, 50% fulfilled modification of chromatin or DNA. Herein, pathways of Wnt/β-catinin signaling, cell cycle-G1/S check point regulation, TGF-β signaling and apoptosis were exploited, including TGF-β, c-MYC, FOXH1, FOXK2, FOXH1, CCNE1, SMARCC1, HTRA2, DIABLO, ICAD, FZD2, CTBP1, ACTR1A, CHD1L, TLE3, PPP2R5D, SMARCD2 and HDAC1 and so forth.
