# Supplementary material for: Relationships between Hematopoiesis and Hepatogenesis in the Midtrimester Fetal Liver Characterized by Dynamic Transcriptomic and Proteomic Profiles
Source: PLoS One. 2009 Oct 28;4(10):e7641. doi: 10.1371/journal.pone.0007641 (PMC2765071; doi:10.1371/journal.pone.0007641)
Supplement: Table S1 — Differentially expressed protein spots for mouse livers of the four different developmental stages (0.03 MB DOC) [file pone.0007641.s007.doc]

**Table S1** Differentially expressed protein spots for mouse livers of the four different developmental stages

| The comparative pairs (two gestational ages) | Amount of differentially expressed proteins spots* | Absolute amount of differentially expressed protein spots** |
| --- | --- | --- |
| E11.5/E14.5 | 63 | 133 |
| E14.5/E15.5 | 37 |
| E11.5/E15.5 | 113 |
| E11.5/3d | 399 | 585 |
| E14.5/3d | 378 |
| E15.5/3d | 381 |
| Total spots |  | 611 |
| Spots identified |  | 328 |
| Genes identified |  | 187 |

*student t test：＞2.0 fold, p＜0.05; **The redundant spots were removed
