# Supplementary material for: Relationships between Hematopoiesis and Hepatogenesis in the Midtrimester Fetal Liver Characterized by Dynamic Transcriptomic and Proteomic Profiles
Source: PLoS One. 2009 Oct 28;4(10):e7641. doi: 10.1371/journal.pone.0007641 (PMC2765071; doi:10.1371/journal.pone.0007641)
Supplement: Table S4 — Components of protein-ubiquitination pathway emerged in type A and B (0.03 MB DOC) [file pone.0007641.s010.doc]

**Table S4** Components of protein-ubiquitination pathway emerged in type A and B

|  | E1 | E2 (class*) | E3 (class**) | Proteasome  subunits | DUB | Ancillary  proteins |
| --- | --- | --- | --- | --- | --- | --- |
| Type A | UBE1L2 | UBE2V2 (Ⅰ) UBE2O (Ⅳ) | UBE3A (HECT) DZIP3 (RING) | PSMC6 | UCHL5 UCH37 USP1 USP33 USP46 |  |
| Type B | SAE1 SAE2 | UBE2I (Ⅰ) UBE2M (Ⅰ) UBE2N (Ⅰ) UBE2G2 (Ⅰ) | RNF26 (RING) RNF187 (RING) TRIM27 (RING) SUGT1 (SCF) SKP2^#^ (SCF) ANAPC10 (APC) CDC23 (APC) CDC16 (APC) | PSMB5 PSMC3 PSMC4 PSMD3 PSMD4 | BAP1 UCHL3 USP10 | HSPA8 |

****:*** *Four classes of E2 enzymes: class I enzymes consist of just the catalytic core domain (UBC), class II possess a UBC and a C-terminal extension, class III possess a UBC and an N-terminal extension, and class IV possess a UBC and both N- and C-terminal extensions.*

*****:*** *The classes of E3s: HECT E3s (homologous to E6-associated protein C-terminus), RING E3s (really interesting new gene), U-box* *E3s (a modified RING motif without the full complement of Zn^2+^-binding ligands) and multi-complex E3s (SCF: Skp1–Cullin 1(Cul1)–F-boxproteins, APC: anaphase-promoting complex).*

*#: SKP2 is also considered to be a SCF cofactor(Reed, 2006).*
