# Supplementary material for: Relationships between Hematopoiesis and Hepatogenesis in the Midtrimester Fetal Liver Characterized by Dynamic Transcriptomic and Proteomic Profiles
Source: PLoS One. 2009 Oct 28;4(10):e7641. doi: 10.1371/journal.pone.0007641 (PMC2765071; doi:10.1371/journal.pone.0007641)
Supplement: Table S5 — Experimental design of DIGE. Mice livers of three stages, E11.5, E14.5, E15.5 and 3 dpp, For each sample pair, triplicate gels were run, which are represented by number 1, 2 and 3. (0.03 MB DOC) [file pone.0007641.s011.doc]

**Table S5. *Experimental design of DIGE*. Mice livers of three stages, E11.5, E14.5, E15.5 and 3 dpp, For each sample pair, triplicate gels were run, which are represented by number 1, 2 and 3.**

| Gel | Cy2 | Cy3 | Cy5 |
| --- | --- | --- | --- |
| 1 | Pool of all samples | E11.5 | E14.5 |
| 2 | Pool of all samples | E11.5 | E14.5 |
| 3 | Pool of all samples | E11.5 | E14.5 |
| 4 | Pool of all samples | E15.5 | 3dpp |
| 5 | Pool of all samples | E15.5 | 3dpp |
| 6 | Pool of all samples | E15.5 | 3dpp |
