# Supplementary material for: Relationships between Hematopoiesis and Hepatogenesis in the Midtrimester Fetal Liver Characterized by Dynamic Transcriptomic and Proteomic Profiles
Source: PLoS One. 2009 Oct 28;4(10):e7641. doi: 10.1371/journal.pone.0007641 (PMC2765071; doi:10.1371/journal.pone.0007641)
Supplement: Table S6 — PCR Primers and PCR Conditions (0.06 MB DOC) [file pone.0007641.s012.doc]

**Table S6. PCR Primers and PCR** Conditions

| Gene Name | Primer name | Sequence (5'→ 3') | Annealing Temperature (°C) | Product Sizes (bp) |
| --- | --- | --- | --- | --- |
| KITL | forward | acgtggaccagtggaagaac | 55 | 344 |
|  | reverse | tccgagtccttgcattcttt |  |  |
| ITGA4 | forward | gcctactgtctcggcagaat | 55 | 172 |
|  | reverse | acagctttccctgtcttgga |  |  |
| ADRB2 | forward | tgttggttctggaggactga | 55 | 185 |
|  | reverse | tcacagcagaaaggtccaag |  |  |
| ZFX | forward | ccctgaacaagtgctggatt | 55 | 262 |
|  | reverse | ggcacagtctgctaccaaca |  |  |
| PRDM16 | forward | caccctcaacacctccactt | 55 | 108 |
|  | reverse | tgaggccagttctgagaggt |  |  |
| KLF6 | forward | ggcctctctttggaaggtct | 55 | 177 |
|  | reverse | aaccatcccacccactaaca |  |  |
| FOXA1 | forward | tacccacctacgctggaaat | 55 | 287 |
|  | reverse | gccaagaataggatggcaag |  |  |
| RUNX1 | forward | cggttcctaccagttctcca | 56 | 309 |
|  | reverse | agtttccctccgggattctt |  |  |
| ACOX1 | forward | gccttgacctctgatcctca | 55 | 155 |
|  | reverse | ctgttctcacgatgccaatg |  |  |
| PTGS1 | forward | agaacccagtgtccagcaag | 56 | 127 |
|  | reverse | gagtccatctgttccctcca |  |  |
| KHK | forward | gaggtggtgtttgtcagcaa | 55 | 235 |
|  | reverse | tgacagaggcattgaaggtg |  |  |
| HEXB | forward | catcctttctgctccttggt | 55 | 296 |
|  | reverse | ctattccacggctgaccatt |  |  |
| SMARCA5 | forward | gcagcagccacaagataatg | 56 | 273 |
|  | reverse | ctgcttcagggtctccagtt |  |  |
| SAMD4 | forward | actctgcctgctgcttcact | 56 | 133 |
|  | reverse | gatcaaggatggcaaacaca |  |  |
| CREBBP | forward | caagacctgctacggaccctaaa | 57 | 388 |
|  | reverse | gctgtctcctcaccatttctcg |  |  |
| GAPDH | forward | atgcatcctgcaccaccaactgct | 58 | 356 |
|  | reverse | cctcagatgcctgcttcaccacct |  |  |
